# Damage Detection in Beam Structures Based on Frequency-Domain Analysis Methods for Nonlinear Systems

**DOI:** 10.3390/s25092901

**Published:** 2025-05-04

**Authors:** Wenbo Zhang, Xiaoyue Guo, Liangliang Cheng, Bo Zhang

**Affiliations:** 1 Dynamics and Vibration Group, Engineering and Technology Institute Groningen, Faculty of Science and Engineering, University of Groningen, 9747 AG Groningen, The Netherlands; w.zhang@rug.nl; 2 School of Mechanical Engineering, Ningxia University, Yinchuan 750021, China; gy188132@163.com

**Keywords:** NOFRFs, GALEs, structural damage detection, NARX model, FRF

## Abstract

Structural damage detection is crucial for ensuring the safety and durability of engineering systems. Conventional detection methods based on the frequency response function (FRF) in linear systems tend to fail when small early damage occurs in engineered structures. Nonlinear output frequency response functions (NOFRFs), which are extensions of the FRF in linear systems to weak nonlinear systems, have been applied in nonlinear system analysis. In this study, we extended the structural damage detection method based on NOFRFs to multi-degree-of-freedom systems and beam structures. Due to the presence of multiple modal frequencies in these structures, the nonlinear characteristic frequencies exhibited by the system are often more complex than those of typical rotor systems, significantly increasing the difficulty of system identification and the feasibility of frequency-domain analysis. To improve the accuracy of the Nonlinear Auto-Regressive with eXogenous inputs (NARX) model and reduce the impact of noise interference, we proposed a Multi-input Multi-output Forward Regression Orthogonal Least Squares (MFROLS) algorithm for processing multi-input multi-output data to identify the NARX model of the same structural system. Next, a numerical simulation study was conducted using the combined NARX model and Generalized Associated Linear Equations (GALEs) method, taking a one-dimensional multi-degree-of-freedom (MDOF) system as an example. Nonlinear stiffness terms were introduced into the MDOF system to simulate structural damage, and a comparative study was performed with a least squares method (LSM). The results show that the proposed method can capture the trends of dynamic characteristic changes in the one-dimensional MDOF system under the influence of different nonlinear stiffnesses, whereas the LSM fails to do so. Finally, experimental research was carried out on simply supported beams with varying degrees of damage. The results demonstrate that the frequency-domain analysis method based on nonlinear systems can detect differences in damage levels in beam structures, providing a new approach for structural damage detection.

## 1. Introduction

In engineering practice, the integrity and longevity of structures and machines are crucial. Detecting and assessing damage is essential for maintaining safety, efficiency, and performance. Consequently, researchers have extensively studied and proposed various structural damage detection techniques. Among these, vibration-based methods have gained significant attention. These methods treat structures as mechanical systems defined by mass, damping, and stiffness matrices. When cracking damage occurs, changes in physical parameters, such as frequency [1], vibration pattern, curvature modes [2,3], and modal flexibility (stiffness) [4], alter the system’s modal parameters and response characteristics. Monitoring these changes enables the assessment of damage presence and extent. Vibration analysis methods based on modal correlation parameters are inherently linear. However, traditional linear damage detection methods struggle to address the complexities of nonlinear behavior in mechanical systems under dynamic loads. American scholars Farrar and Doebling [5] believe that modal parameters reflect the system’s global characteristics, while damage is a local phenomenon with minimal impact on overall modal parameters, indicating their insensitivity to localized damage. Experiments by Chen and Spyrakos [6] show that even cracks severe enough to cause beam failure result in less than a 5% change in the first four natural frequencies. Additionally, due to prevalent nonlinearities, modal test results vary with excitation strength, affecting modal frequencies and shapes [7]. To address this, nonlinear vibration-based methods [8,9,10,11] have been developed, detecting damage by extracting features like super-harmonics and sub-harmonics. Compared to linear methods, nonlinear approaches are more promising for structural damage detection. However, most current nonlinear detection methods are based on signal analysis techniques, which extract nonlinear frequencies from signals to research (e.g., using nonlinear Lamb waves for damage detection in plate structures). But these methods often fail to exclude the influence of nonlinearities inherent in the excitation signals themselves, as they do not analyze the structural nonlinear dynamic behavior from the perspective of the system itself. The FRF is a powerful tool for describing the dynamic behavior of linear systems, and this concept has since been extended to nonlinear systems.

The dynamic properties of nonlinear systems are more complex than those of linear systems, which can be simply described by the frequency response function (FRF). The FRF is a fundamental concept in linear systems, serving as the theoretical basis for classical control and structural system design and analysis and is widely used in engineering practice [12]. Leveraging the advantages of the FRF in linear system analysis, scholars have extended this concept to nonlinear systems using Volterra series theory, introducing generalized frequency response functions (GFRFs) and nonlinear output frequency response functions (NOFRFs) [13]. NOFRFs were proposed in [13] as an extension of the FRF to nonlinear systems, enabling simplified analysis using Bode-like diagrams while retaining the ability to fully describe nonlinearities [14]. In some cases, NOFRFs can directly indicate the strength of nonlinearities [10] and have been successfully applied in fault detection and control of various nonlinear systems. Typically, NOFRFs are computed using the least squares method (LSM), which requires multiple system excitations, making real-time monitoring and control challenging and introducing truncation errors. To address these issues, Zhu et al. [15] proposed generalized associated linear equations (GALEs). Therefore, from the perspective of studying the system itself, employing nonlinear system frequency-domain analysis methods for structural damage detection offers distinct advantages.

**Figure 1:**
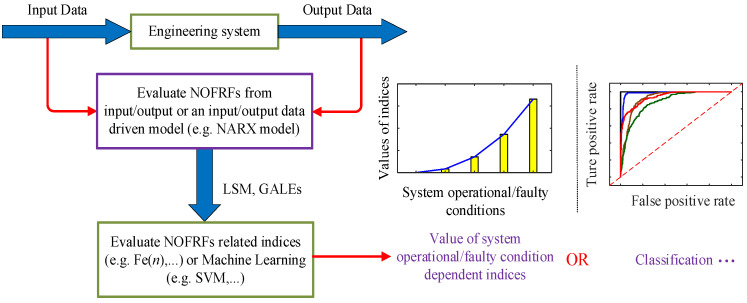
Condition monitoring or fault diagnosis of engineering systems based on NOFRFs.

In practical engineering, structural damage detection methods based on data-driven models (e.g., NARX models) are more versatile due to system complexity and sensor signal discretization. Figure 1 illustrates the NOFRF-based framework for condition monitoring or fault diagnosis in engineering systems. Identify the nonlinear model from the engineering system data, then use the nonlinear frequency-domain analysis method to obtain the NOFRFs describing the system dynamics, and finally choose the appropriate nonlinear index Fe(n) or use and machine learning methods (such as support vector machine, SVM) for classification and index judgment. However, most NOFRFs-based structural damage detection methods have primarily targeted rotor systems exhibiting typical nonlinear behaviors. Since rotor systems predominantly generate harmonic signals with relatively distinct frequency-doubling phenomena, modeling efforts could focus exclusively on specific frequency components. In this study, we extend the NOFRFs-based damage detection methodology to MDOF systems and beam structures. The presence of multiple modal frequencies in these structures results in significantly more complex nonlinear characteristic frequencies compared to conventional rotor systems, substantially increasing the challenges of system identification and the feasibility of frequency-domain analysis. To enhance the accuracy of NARX models and mitigate noise interference, we propose an MFROLS algorithm for identifying NARX models of such structural systems. Subsequently, numerical simulations were conducted on a one-dimensional MDOF system using a combined NARX-GALEs approach. By introducing nonlinear stiffness terms to simulate structural damage and comparing the results with the LSM, our method demonstrated that the method based on the one proposed in this paper is able to capture the trend of the dynamical characteristics of a one-dimensional multi-degree-of-freedom system under the influence of different nonlinear stiffnesses, whereas the LSM is not able to do so. Finally, an experimental study was carried out with simply supported beams with different degrees of damage as an example, and the results show that the method based on frequency-domain analysis of nonlinear systems can detect the different changes in degrees of damage in the beam structure, which provides a new way of thinking for structural damage detection.

## 2. Materials and Methods

### 2.1. Frequency Response Function of the Linear Systems

When studying linear systems, the Auto-Regressive with eXogenous inputs (ARX) model, a widely used linear dynamic system model, is extensively applied in system identification, signal processing, and control engineering. It enables the construction of a dynamic mathematical model of the system based on its input and output data.

(1) $$y(k)=-a_{1}y(k-1)-a_{2}y(k-2)-\cdots -a_{n}y(k-n)+b_{0}u(k-1)+\cdots +b_{m}u(k-m)+e(k)$$

where *y*(*k*) represents the output, *u*(*k*) denotes the input, *e*(*k*) signifies the noise, and $a_{i}$ and $b_{i}$ are the model parameters. For a linear system with an input *u*(*k*) and an output *y*(*k*) in the discrete time, researchers often use a convolution integral expression to represent the mathematical relationship between the system’s input and output in the time domain.

(2) $$y(k)=\sum_{\tau =-\infty }^{+\infty }h(\tau )u(k-\tau )$$

where *h*(*τ*) is the impulse response function of the linear system.

When we study the dynamic characteristics of a linear system in the frequency domain, the relationship between the frequency-domain input and the frequency-domain output of the linear system can be expressed as follows:

(3) Y(jω)=H(jω)U(jω)

where *Y*(j*ω*) and *U*(j*ω*) represent the Fourier transform of the system’s time-domain input *u*(*k*) and output *y*(*k*). *H*(j*ω*) is the form of the system’s impulse response function *h*(*τ*) after Fourier transformation, which can also be referred to as the Frequency Response Function (FRF). In control engineering, this is also referred to as the transfer function. As shown in Equation (3), the output frequency composition remains identical to that of the input, demonstrating that the linear characteristics of the system do not alter the input frequency components.

### 2.2. Nonlinear Output Frequency Response Functions of Nonlinear Systems

In the study of nonlinear systems, a mathematical model can be selected to characterize the system under investigation. In practice, a general class of nonlinear systems can be effectively represented by a polynomial NARX model [16].

(4) $$y(k)=\sum_{m=1}^{M}\sum_{\begin{array}{l} p=0;\\ p+q=m \end{array}}^{m}\sum_{k_{1},\;k_{p+q}=1}^{K}C_{p,q}(k_{1},\cdots ,k_{p+q})\prod_{i=1}^{p}y(k-k_{i})\prod_{i=p+1}^{p+q}u(k-k_{i})$$

where *M* represents the maximum order of nonlinearity; *K* is the maximum time delay; and $C_{p,q}(l_{1},\ldots ,l_{p+q})$ are the coefficients of model Equation (4).

(5) $$\sum_{k_{1},\;k_{p+q}=1}^{K}=\sum_{k_{1}=1}^{K}\cdots \sum_{k_{p+q}=1}^{K}\;$$

For nonlinear systems in the discrete-time domain, such as the NARX model described by Equation (4), if the system is asymptotically stable at the zero equilibrium point, then the system’s output *y*(*k*) can be represented by a discrete-time Volterra series as follows:

(6) $$y(k)=\sum_{n=1}^{+\infty }y_{n}(k)\approx \sum_{n=1}^{N}\sum_{\tau_{1}=-\infty }^{+\infty }\cdots \sum_{\tau_{n}=-\infty }^{+\infty }h_{n}(\tau_{1},\tau_{2},\cdots ,\tau_{n})\prod_{i=1}^{n}u(k-\tau_{i})$$

In the frequency domain, the output spectrum of the system *Y*(j*ω*) can be expressed as

(7) $$Y(j\omega )=\sum_{n=1}^{N}Y_{n}(j\omega )=\sum_{n=1}^{N}\frac{1/\sqrt{n}}{{\left(2\pi \right)}^{n-1}}\int_{\omega_{1}+\omega_{2}+\cdots +\omega_{n}=\omega }H_{n}(j\omega_{1},j\omega_{2},\cdots ,j\omega_{n})\prod_{i=1}^{n}U(j\omega_{i})d\delta \omega$$

where $Y_{n}$(j*ω*) and $U_{n}$(j*ω*) are the nth order spectra of output and input obtained from the Fourier Transform of $y_{n}$(*k*) and $u_{n}$(*k*), respectively, and $H_{n}(j\omega_{1},\ldots ,j\omega_{n})$ is the generalized frequency response function of the system. Based on Equation (7), Lang and Billings [13] proposed a new concept in the frequency domain, the nonlinear output frequency response function, which is defined as:

(8) $$\begin{array}{l} G_{n}(j\omega )=\frac{\frac{1/\sqrt{n}}{{\left(2\pi \right)}^{n-1}}\int_{\omega_{1}+\omega_{2}+\cdots +\omega_{n}=\omega }H_{n}(j\omega_{1},j\omega_{2},\cdots ,j\omega_{n})\prod_{i=1}^{n}U(j\omega_{i})d\delta \omega }{\frac{1/\sqrt{n}}{{\left(2\pi \right)}^{n-1}}\int_{\omega_{1}+\omega_{2}+\cdots +\omega_{n}=\omega }\prod_{i=1}^{n}U(j\omega_{i})d\delta \omega }\\ \;=\frac{\int_{\omega_{1}+\omega_{2}+\cdots +\omega_{n}=\omega }H_{n}(j\omega_{1},j\omega_{2},\cdots ,j\omega_{n})\prod_{i=1}^{n}U(j\omega_{i})d\delta \omega }{\int_{\omega_{1}+\omega_{2}+\cdots +\omega_{n}=\omega }\prod_{i=1}^{n}U(j\omega_{i})d\delta \omega }\;=\frac{Y_{n}(j\omega )}{U_{n}(j\omega )},\;\omega \in \Omega \end{array}$$

where $\int_{\omega_{1}+\omega_{2}+\cdots +\omega_{n}=\omega }\prod_{i=1}^{n}U(j\omega_{i})d\delta \omega \neq 0$. After introducing the nonlinear output frequency response function $G_{n}$(j*ω*) for *n* = 1, …, *N*, the output spectrum of the system can be represented by the following formula:

(9) $$Y(j\omega )=\sum_{n=1}^{N}Y_{n}(j\omega )=\sum_{n=1}^{N}G_{n}(j\omega )U_{n}(j\omega )$$

The output spectrum of the nonlinear system represented by Equation (9) is similar to that of a linear system, where $G_{n}$(j*ω*) for *n* ≥ 1 is the NOFRFs defined as follows:

(10) $$G_{n}(j\omega )=\frac{Y_{n}(j\omega )}{U_{n}(j\omega )},\;\omega \in \Omega_{n}$$

where $\Omega_{n}$ is the frequency support of *|*$U_{n}$(j*ω*)*|*.

### 2.3. Identification and Validation of the NARX Model

Various methods exist for identifying NARX models in nonlinear systems, such as the Orthogonal Least Squares (OLS) algorithm [17], Regularization Forward Regression Orthogonal Least Squares (RFROLS) algorithm [18], Least Absolute Shrinkage and Selection Operator (LASSO) algorithm [19], and Least Angle Regression (LAR) algorithm [20]. Among these, the MFROLS (Modified Forward Regression with Orthogonal Least Squares) algorithm, an advanced extension of the FROLS algorithm proposed by Stephen Billings [21], is particularly effective for complex multivariate systems. MFROLS improves upon FROLS by efficiently modeling relationships between multiple inputs and outputs, making it suitable for NARX identification problems with shared model structures but differing coefficients. After identifying the NARX model using the MFROLS algorithm, model validation is essential to ensure its accuracy. Two primary methods are commonly used for NARX model validation: (1) the One Step Ahead (OSA) prediction; (2) the Model Prediction Output (MPO).

For example, the NARX model for a system is expressed as

(11) $$y(k)=a_{1}y(k-1)+a_{2}y(k-2)+b_{1}u(k-1)$$

Then, the predicted output response of the OSA model of the system is

(12) $$\left\{\begin{array}{l} \hat{y}(1)=y(1)\\ \hat{y}(2)=y(2)\\ \hat{y}(3)={\hat{a}}_{1}y(2)+{\hat{a}}_{2}y(1)+{\hat{b}}_{1}u(2)\\ \vdots \\ \hat{y}(k)={\hat{a}}_{1}y(k-1)+{\hat{a}}_{2}y(k-2)+{\hat{b}}_{1}u(k-1) \end{array}\right.$$

where y denotes the actual output of the NARX model, $\hat{y}$ denotes the predicted output of the NARX model, and ${\hat{a}}_{1},{\hat{a}}_{2},{\hat{b}}_{1}$ denote the model coefficients.

While in MPO model prediction, the input of the later step is the model prediction output of the previous step, and the prediction error accumulates gradually, the MPO model prediction output of NARX in Equation (12) is

(13) $$\left\{\begin{array}{l} \hat{y}(1)=y(1)\\ \hat{y}(2)=y(2)\\ \hat{y}(3)={\hat{a}}_{1}\hat{y}(2)+{\hat{a}}_{2}\hat{y}(1)+{\hat{b}}_{1}u(2)\\ \vdots \\ \hat{y}(k)={\hat{a}}_{1}\hat{y}(k-1)+{\hat{a}}_{2}\hat{y}(k-2)+{\hat{b}}_{1}u(k-1) \end{array}\right.$$

From the above analysis, it is evident that in MPO, the output at each subsequent step is derived from the model’s predicted output in the previous step, leading to the gradual accumulation of prediction errors. While OSA demonstrates the model’s fitting capability, MPO rigorously evaluates its predictive performance, providing a stringent assessment of whether the NARX model accurately captures the dynamics of the actual system. Before evaluating the NOFRFs, it is essential to validate the identified NARX model. Traditional NOFRFs-based fault diagnosis methods rely on the Least Squares (LS) algorithm, which requires multiple tests with varying excitation amplitudes [21]. In contrast, the recently developed GALEs [15] enable direct evaluation of NOFRFs for NARX models. Detailed steps for calculating GALEs are provided in Appendix A.

## 3. Structural Damage Detection Based on NARX Model

### 3.1. Dynamic Modeling of One-Dimensional Multi-Degree-of-Freedom Systems

**Figure 2:**
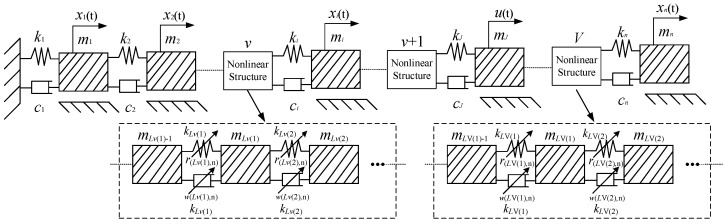
One-dimensional MDOF systems.

Figure 2 illustrates a one-dimensional multi-degree-of-freedom (MDOF) system, where the input excitation is applied to the *j*-th mass block.

If all the stiffnesses and damping of system are linear, and the dynamic equations of the MDOF system are

(14) $$M\ddot{x}+C\dot{x}+Kx=F(t)$$

where **M**, **C**, and **K** denote the mass matrix, damping matrix, and stiffness matrix of the MDOF system, respectively; **F** denotes the system input excitation vector; and **x** is the displacement vector of the system.

(15) $$\begin{array}{c} M=\left[\begin{array}{cccc} m_{1} & 0 & \cdots & 0\\ 0 & m_{2} & \cdots & 0\\ \vdots & \vdots & \ddots & \vdots \\ 0 & 0 & \cdots & m_{n} \end{array}\right],\;C=\left[\begin{array}{ccccc} c_{1}+c_{2} & -c_{2} & 0 & \cdots & 0\\ -c_{2} & c_{2}+c_{3} & -c_{3} & \ddots & \vdots \\ 0 & \ddots & \ddots & \ddots & \vdots \\ \vdots & \ddots & -c_{n-1} & c_{n-1}+c_{n} & -c_{n}\\ 0 & 0 & 0 & -c_{n} & c_{n} \end{array}\right],\;x=\left[\begin{array}{c} x_{1}\\ \vdots \\ x_{n} \end{array}\right],\\ K=\left[\begin{array}{ccccc} k_{1}+k_{2} & -k_{2} & 0 & \cdots & 0\\ -k_{2} & k_{2}+k_{3} & -k_{3} & \ddots & \vdots \\ 0 & \ddots & \ddots & \ddots & \vdots \\ \vdots & \ddots & -k_{n-1} & k_{n-1}+k_{n} & -k_{n}\\ 0 & 0 & 0 & -k_{n} & k_{n} \end{array}\right],\;F(t)={\left[\begin{array}{c} \overset{J-1}{\overset{︷}{\begin{array}{ccc} 0 & \cdots & 0 \end{array}}}\;u(t)\;\overset{n-J}{\overset{︷}{\begin{array}{ccc} 0 & \cdots & 0 \end{array}}} \end{array}\right]}^{T} \end{array}$$

When a nonlinear spring or nonlinear damping is present in the system, the dynamical equations for the MDOF system of Figure 2 are

(16) $$M\ddot{x}+C\dot{x}+Kx=F(t)+NF(t)$$

Assuming the MDOF system in Figure 2 contains *V* nonlinear structural components, where each component consists of one or more continuous nonlinear elements (nonlinear springs or dampers). Each nonlinear component is labeled as *v* and numbered from left to right as *v* = 1, …, *V* (with *V < n)*. The number of nonlinear elements in each component is denoted as $\bar{L}v$, where $\bar{L}V<n$. The nonlinear springs in each component are labeled from left to right as *Lv*(*i*), where $i=1,\cdots ,\bar{L}v$, and *Lv*(*i*) correspond to the index of the mass block to the right of the nonlinear element in that component. Assuming the highest order of nonlinearity in the system’s stiffness and damping is $\bar{N}$, the nonlinear force generated by each nonlinear element can be expressed as

(17) $$\left\{\begin{array}{l} f_{s}(i)=\sum_{\bar{n}=2}^{\bar{N}}r_{\left(Lv(i),\bar{n}\right)}{\left(x_{Lv(i)}(t)-x_{Lv(i)-1}(t)\right)}^{\bar{n}}\\ f_{d}(i)=\sum_{\bar{n}=2}^{\bar{N}}w_{\left(Lv(i),\bar{n}\right)}{\left({\dot{x}}_{Lv(i)}(t)-{\dot{x}}_{Lv(i)-1}(t)\right)}^{\bar{n}} \end{array}\right.,\;i=1,\cdots ,\bar{L}v,\;v=1,\cdots ,V<n$$

where $f_{s}(i)$ and $f_{d}(i)$ represent the nonlinear elastic restoring force and the nonlinear damping force produced by the nonlinear element, respectively. $r_{\left(Lv(i),\bar{n}\right)}$ and $w_{\left(Lv(i),\bar{n}\right)}$ represent the nonlinear stiffness and nonlinear damping of the *i*th nonlinear element in the *v*th part of the nonlinear structure, respectively. The vector of the nonlinear forces produced is

(18) $$Nfv(i)={\left[\begin{array}{cccc} \overset{Lv(i)-2}{\overset{︷}{\begin{array}{ccc} 0 & \cdots & 0 \end{array}}} & fs(i)+fd(i) & -\left(fs(i)+fd(i)\right) & \overset{n-Lv(i)}{\overset{︷}{\begin{array}{ccc} 0 & \cdots & 0 \end{array}}} \end{array}\right]}^{T}$$

The sum of the nonlinear forces generated by part *v* is

(19) $$NFv(t)=\sum_{i=1}^{\bar{L}v}Nfv(i)$$

Then, the nonlinear force of the whole MDOF system consists of the *V*-part nonlinear structure:

(20) $$NF(t)=\sum_{v=1}^{V}Nfv(t)$$

### 3.2. Structural Damage Detection of MDOF Systems with a Single Nonlinear Spring

**Figure 3:**
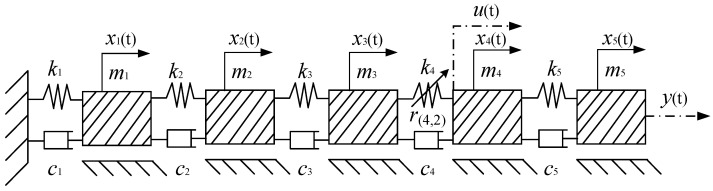
One-dimensional 5-degree-of-freedom system.

In this subsection, structural damage of varying degrees is simulated by introducing a single spring with different nonlinear stiffness values into a one-dimensional 5-degree-of-freedom system. Since the NDE and NARX models, along with their GALEs, are applicable only to Single-Input Single-Output (SISO) systems, the one-dimensional 5-degree-of-freedom system shown in Figure 3, which is a Single-Input Multiple-Output (SIMO) system, requires specific treatment. To address this, the output response $x_{5}$ of the fifth mass block $m_{5}$ is selected as the system’s output. The NARX model identification is then conducted using the input excitation applied to the fourth mass block and the output response from the fifth mass block.

When initial cracks or structural damage occur in practical engineering structures, the original linear constitutive equation of the material transitions into a nonlinear one. This nonlinearity typically originates from the emergence of nonlinear stiffness in the structure, making nonlinear stiffness a more universal indicator for simulating structural damage. Furthermore, due to the current lack of a clear mechanistic understanding of nonlinear damping, our numerical simulations primarily introduce nonlinear stiffness to model structural damage. Figure 3 illustrates a one-dimensional 5-degree-of-freedom system, where *n* = 5. The masses are set as $m_{1}$ = $m_{2}$ = … = $m_{5}\;$= 1 kg, the linear stiffness values are $k_{1}$ = $k_{2}$ = … = $k_{5}$ = $k_{0}$ = 3.6 × $10^{4}$ N/m, and the linear damping coefficients are $c_{1}$ = $c_{2}$= … = $c_{5}$ = 3.6 × $10^{2}$ N/m. The input excitation is applied at position *J* = 4. A nonlinear spring is positioned exclusively between mass blocks 3 and 4, indicating *V =* 1, $\bar{L}v=\bar{L}1=1$, *Lv*(1) = *L*1(1) = 4. The highest order of nonlinear stiffness in the system is *N* = 2, and no nonlinear damping terms are present, that is, $w_{\left(Lv(i),\bar{n}\right)}=0$. The nonlinear stiffness of the system is expressed as follows:

(21) $$r_{\left(Lv(i),\bar{n}\right)}=r_{\left(4,2\right)}=\left[0,0.01,0.1,0.2,0.3,0.4,0.5,0.6,0.7\right]k_{0}^{2}$$

The specific steps for structural damage detection based on the NARX model are given below according to the ideas in Figure 1, where the linear MDOF system ($r_{\left(4,2\right)}$ = 0) and the nonlinear MDOF system ($r_{\left(4,2\right)}$ = 0.1*k*_0_^2^) are illustrated as examples, respectively.

- Step 1: Exposure to systematic excitation:

Add two separate Gaussian random excitations to both the linear MDOF system denoted by Equation (14) and the nonlinear MDOF system denoted by Equation (16) and use the respective two random excitations as the input signals to the system, denoted as $u_{a}(t)$ and $u_{b}(t)$.

- Step 2: Identification of the NARX model:

The output response $x_{5}$ of the mass block $m_{5}$ is selected as the system’s output response y(t) of the system, as shown in Figure 3. The two output responses of the system are computed using the fourth-order Runge–Kutta method, denoted as $y_{a}(t)$ and $y_{b}(t)$. For both states, the NARX model (or ARX model for the linear system) is identified using the MFROLS algorithm described in Section 2.2. The identification results are labeled as NARX-a (or ARX-a) and NARX-b (or ARX-b), respectively. Linear MDOF system identification results (no damage structure $r_{\left(4,2\right)}$ = 0):

(22) $$\begin{array}{l} y(k)=C_{1,0}(1)y(k-1)+C_{1,0}(2)y(k-2)+C_{1,0}(3)y(k-3)+C_{0,1}(1)u(k-1)+\\ \;C_{0,1}(2)u(k-2)+C_{1,0}(5)y(k-5)+C_{0,1}(3)u(k-3)+C_{0,1}(4)u(k-4) \end{array}$$

Nonlinear MDOF system identification results (damage structure $r_{\left(4,2\right)}$ = 0.1*k*_0_^2^)

(23) $$\begin{array}{l} y(k)=C_{1,0}(1)y(k-1)+C_{1,0}(2)y(k-2)+C_{1,0}(3)y(k-3)+C_{1,0}(4)y(k-4)+\\ \;C_{1,0}(5)y(k-5)+C_{0,1}(1)u(k-1)+C_{0,1}(2)u(k-2)+C_{0,1}(3)u(k-3)+\\ \;C_{0,1}(4)u(k-4)+C_{1,1}(5,1)y(k-5)u(k-1)+C_{2,0}(5,5)y(k-5)y(k-5)+\cdots \end{array}$$

**Figure 4:**
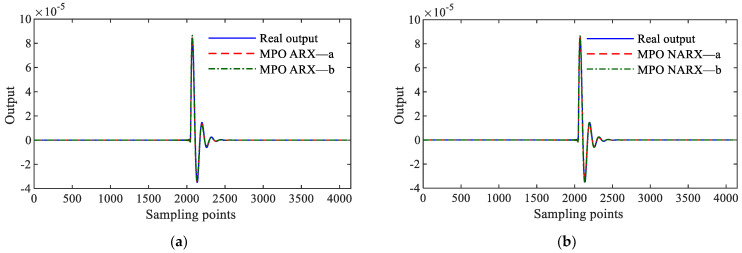
MPO for different MDOF system identification results. (**a**) MPO results for linear MDOF system; (**b**) MPO results for nonlinear MDOF system.

- Step 3: Model validation:

A. The validity of the discriminative model is verified using the MPO prediction validation method described in Section 2.2. Figure 4 presents the MPO predictions for the ARX and NARX models of the discriminative MDOF system (16) under shock excitations.

To evaluate the predictive capability of the identified model’s MPO (Model-Predicted Output), the Variance Accounted For (VAF) is introduced as a common method. The formula for calculating VAF is typically as follows:

(24) $$\left\{\begin{array}{l} \mathrm{VAF}=\left(1-\frac{SS_{res}}{SS_{tot}}\right)\times 100\%\\ SS_{res}=\frac{1}{N}\sum_{k=1}^{N}{\left(y(k)-\hat{y}(k)\right)}^{2},\;SS_{tot}=\frac{1}{N}\sum_{k=1}^{N}y^{2}(k) \end{array}\right.$$

where $SS_{res}$ represents the residual sum of squares, reflecting the prediction error of the model; $SS_{tot}$ represents the total sum of squares, reflecting the variability of the actual data; and *N* represents the data length. As shown in Table 1, the VAF values exceed 95%, confirming the validity of both identification results.

Although the predicted outputs of the two identified ARX models are similar, it is essential to select the appropriate input excitation based on actual engineering application conditions. In this study, a shock excitation with a frequency range of 0 to 80 Hz is chosen. If the identified system exhibits significant differences in VAF under different reference inputs, priority should be given to the input excitation of interest (e.g., the shock excitation in this chapter). The selection is based on the VAF values of the MPO (e.g., ARX-a: 98.92% < ARX-b: 99.23%). Consequently, ARX-b is selected as the final identified result, as the NOFRFs of the system are subsequently calculated using the theory of NARX model GALEs.

B. The validity of the discriminative model is verified using the MPO prediction validation method described in Section 2.2. Figure 4b illustrates the MPO predictions for the NARX-a and NARX-b models of the discriminative nonlinear MDOF system (23) under various excitations.

As shown in Table 1, consistent with the analytical approach above (e.g., NARX-a: 99.30% < NARX-b: 99.37%), NARX-b is chosen as the final identification result for calculating the NOFRFs of the system.

- Step 4: Calculate GALEs for the NARX model:

Although it is possible to compute the GALEs for each order of the system using recursive expressions, this approach involves extensive and complex symbolic calculations, making it highly inefficient. Fortunately, Zhu significantly improved the efficiency of deriving the GALEs for each order of the system. This paper utilizes this advanced algorithm to derive the GALEs, and further details can be found in [22].

- Step 5: Calculate the NOFRFs of the system:

From Equation (16), the five natural frequencies of the system all fall within the range of 0 to 60 Hz. Therefore, in this chapter, a shock signal with a bandwidth of 80 Hz is employed as the input excitation to calculate the NOFRFs of the system at each order.

(25) $$u(k)=\frac{0.02\sin\left(160\pi (k\Delta t-2)\right)}{\pi (k\Delta t-2)},\;k\in [0,\frac{4}{\Delta t}]$$

where Δt is the numerical calculation step, Δt=1/1024.

**Figure 5:**
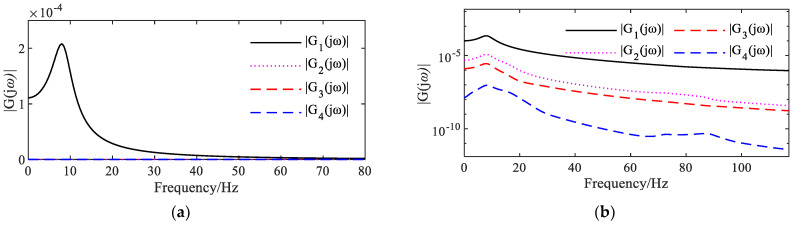
First four orders of NOFRFs for MDOF systems: (**a**) first four orders of NOFRFs for linear systems; (**b**) first four orders of NOFRFs for nonlinear systems.

The nth-order NOFRFs can be obtained from the Fourier transform of the nth-order nonlinear output response $y_{n}(k)$ of the system and the Fourier transform of the input excitation $u_{n}(k)$. Figure 5a represents the first four orders of NOFRFs for the linear MDOF system. Figure 5b shows the first four NOFRFs (Nonlinear Operational Frequency Response Functions) of a nonlinear MDOF (multi-degree-of-freedom) system with *r* = 0.7*k*_0_^2^.

- Step 6: System condition assessment:

In practical engineering, most physical systems are inherently nonlinear, with linear systems typically representing simplified approximations of the actual problem. To visually evaluate the system’s state, this subsection employs the nonlinear stiffness described in Equation (21) to characterize the various damage states of the MDOF system. The aforementioned steps 1 through 5 are repeated to compute the first four orders of NOFRFs for each system under different states.

**Figure 6:**
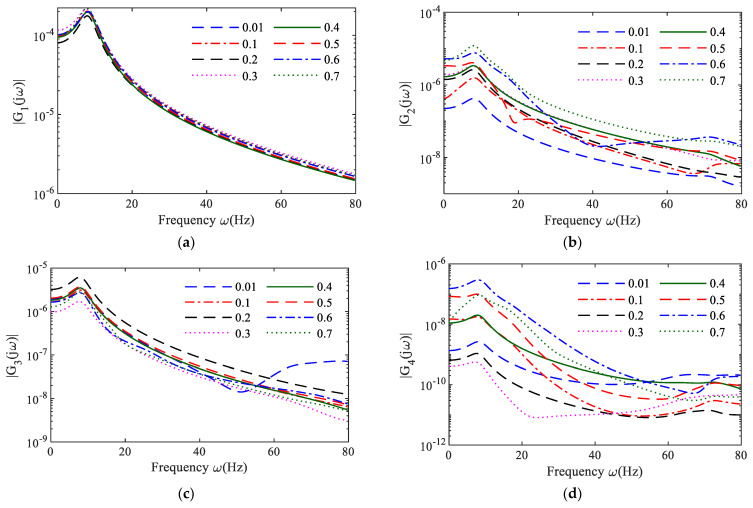
First four orders of NOFRFs for nonlinear MDOF systems using GALEs: (**a**) GALEs-first-order NOFRFs; (**b**) GALEs-second-order NOFRFs; (**c**) GALEs-third-order NOFRFs; (**d**) GALEs-fourth-order NOFRFs.

Figure 6 represents the first four orders of NOFRFs computed by the generalized accompanying linear equations method (GALEs) of the NARX model, respectively, for the MDOF system under different nonlinear stiffnesses.

From Figure 6, it can be observed that, unlike the NOFRFs of simple NDE models (such as the crack beam model represented by a double-linear vibration model), the NOFRFs calculated by the NARX model for the MDOF system under different states (nonlinear stiffness) do not exhibit clear demarcation due to the system’s complexity. As a result, it is not straightforward to directly determine the dynamic state of the system through the nonlinear output frequency response functions of each order. Therefore, in practical engineering applications, it is essential to select appropriate nonlinear indicators for accurate system condition assessment.

**Figure 7:**
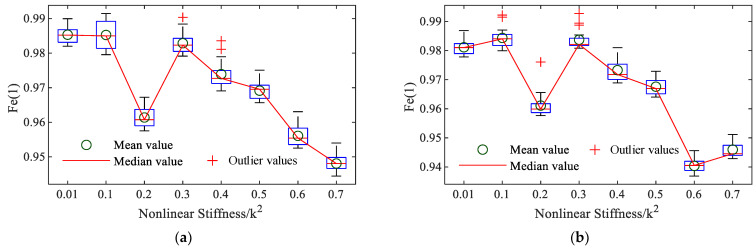

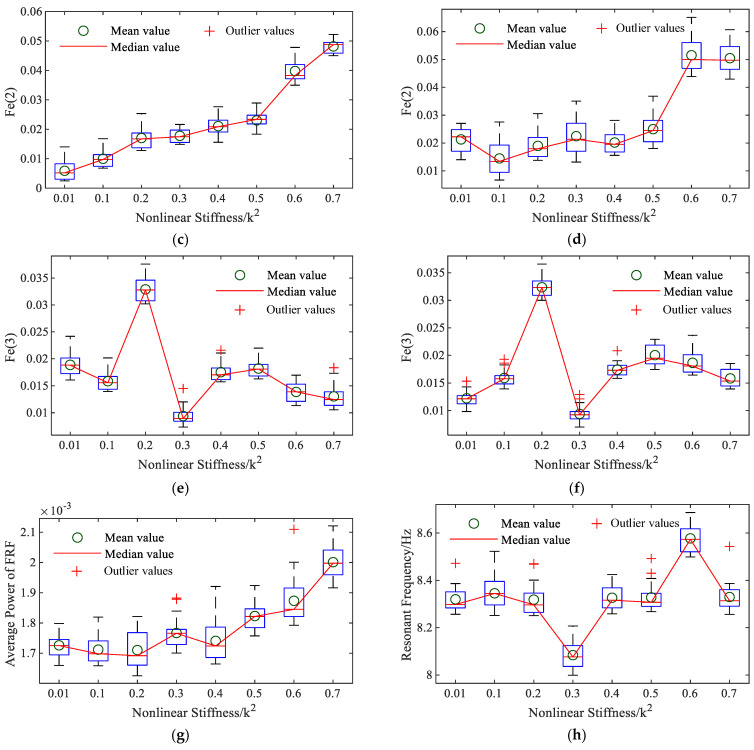
The nonlinear indicators: (**a**) GALEs-Fe(1); (**b**) GALEs-Fe(2); (**c**) LSM-Fe(1); (**d**) LSM-Fe(2); (**e**) GALEs-Fe(3); (**f**) LSM-Fe(3); (**g**) average energy of the system FRF; (**h**) first-order resonant frequency.

Due to the non-uniqueness of the NARX model, to ensure the reliability of the above damage detection method based on the NARX model structure, the identification process of the NARX model for the MDOF system with different nonlinear stiffnesses is repeated 20 times. This results in 20 sets of NOFRFs, and each set of NOFRFs calculated is used for index evaluation. Figure 7 represents the nonlinear indicators obtained under different states from the 20 sets of calculation results. The results obtained from conventional LSM are included for comparison. As demonstrated in this simulation case, the variations in nonlinear stiffness are relatively small, so the conventional LSM does not obtain the system differences caused by these nonlinear stiffness variations. Additionally, a comparison with metrics derived from linear analysis methods further confirms that linear indicators fail to capture these subtle differences induced by nonlinear stiffness variations. Peng [10] pioneered the application of NOFRFs for structural damage detection in aluminum plates and introduced a nonlinear indicator based on NOFRFs, defined as follows:

(26) $$\mathrm{Fe}(n)=\frac{\int_{-\infty }^{+\infty }\left|G_{n}(j\omega )\right|d\omega }{\sum_{i=1}^{N}\int_{-\infty }^{+\infty }\left|G_{i}(j\omega )\right|d\omega },\;1\leq n\leq N$$

where $G_{n}$(j*ω*) is the *n*th order NOFRFs; *N* denotes the highest order of GALEs. Since the above expression is an infinite integral, it can be replaced in applications by an integral over a finite frequency band.

From Figure 7, it can be observed that only the nonlinear indicator Fe(2) in Figure 7c exhibits a monotonic change with increasing nonlinear stiffness. Therefore, Fe(2) can be effectively used to assess the system’s state. To highlight the advantages of structural damage detection based on nonlinear vibration methods, a comparison is made with the method relying on the average power of the FRF and first-order resonant frequency. As shown in Figure 7g,h, these linear approaches are not monotonically related to an increase in nonlinear stiffness (or damage).

## 4. Experimental Research

**Figure 8:**
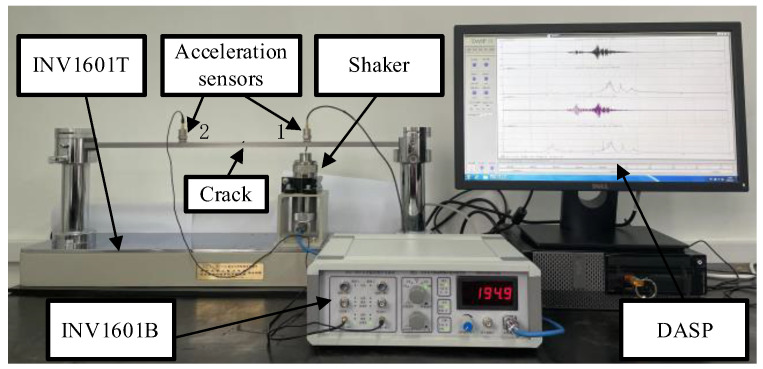
Cracked-beam structural damage detection test bench.

The experimental study utilizes a vibration and control experimental system (type: INV1601) developed by China Orient Institute of Noise & Vibration. This system comprises three main components: (1) vibration teaching experimental bench (type: INV1601T); (2) vibration teaching experimental instrument (type: INV1601B); and (3) vibration teaching experimental software (version: INV1601 DASP). The system is illustrated in Figure 8.

The experimental specimens were designed as beams with geometric dimensions of 700 mm × 50 mm × 10 mm, fabricated from 45-gauge steel. The material properties include a modulus of elasticity E = 206 Gpa, Poisson’s ratio of μ = 0.3, and density of ρ = 7800 kg/m^3^. To simulate varying degrees of structural damage, slots measuring 50 mm in length and 0.2 mm in width were cut at the center of the beams, with depths of 0 mm, 1 mm, 2 mm, 4 mm, and 6 mm. These slots represent five distinct levels of structural damage, labeled as 0, 1, 2, 4, and 6.

**Figure 9:**
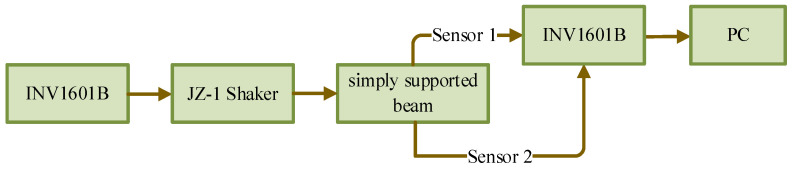
Experimental procedure.

The experimental procedure is illustrated in Figure 9. The process begins with the generation of a swept excitation signal ranging from 10 Hz to 1 kHz, which is produced by the integrated module (INV1601B), incorporating both a power amplifier and function generator. This excitation signal is subsequently applied to the simply supported beam via an electromagnetic shaker (JZ-1). During the experiment, response signals are simultaneously captured by two sensors (Sensor 1 and Sensor 2), with the acquired data being transmitted through the integrated module (INV1601B) to a computer for subsequent processing. The position of the sensors was placed at the left and right third of the beam. This data collection process yields the necessary dataset for NARX model identification. The experimental parameters included a sweep excitation duration of 4 s and a sampling frequency of 1024 Hz.

The following are the specific steps of the structural damage detection scheme based on the NARX model GALEs theory.

**Figure 10:**
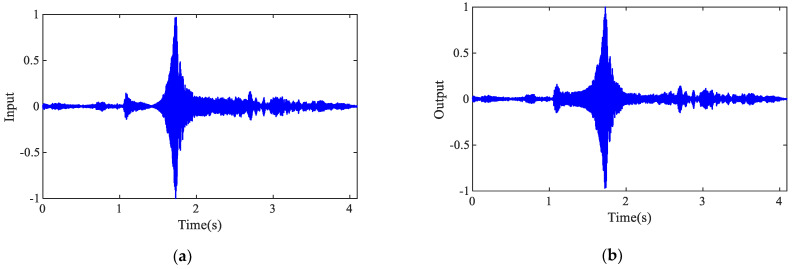
Vibration signals: (**a**) input data; (**b**) output data.

- Step 1: Controlled swept-sine excitation for system identification:

A frequency-sweep excitation signal ranging from 10 Hz to 500 Hz with a 4 s duration was applied to the simply supported beam system using an electromagnetic shaker. The structural responses were simultaneously measured using two accelerometers mounted on the beam surface. Specifically, the acceleration signal acquired from Sensor 1 served as the input dataset for NARX model identification, while the corresponding signal from Sensor 2 was designated as the output dataset. The normalized input–output data pairs, which were subsequently used for system identification, are presented in Figure 10.

The input and output data are collected in two cycles (8 s), and the signal of each cycle is used as a set of input and output data, and finally the NARX model of the system is identified using the MFROLS algorithm.

**Figure 11:**
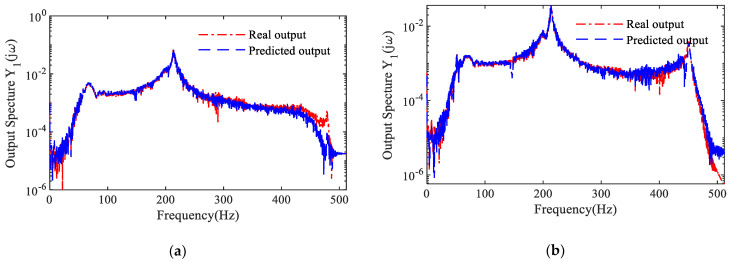
Model prediction output spectrum: (**a**) 0 mm cracked beam; (**b**) 6 mm cracked beam.

- Step 2: Model validation:

The validity of the NARX model is verified through the Model Prediction Output (MPO) method. Figure 11 illustrates the frequency-domain results of the two MPOs for the identified model. Additionally, Table 2 presents the VAF values of the identified NARX models corresponding to different crack depths.

**Figure 12:**
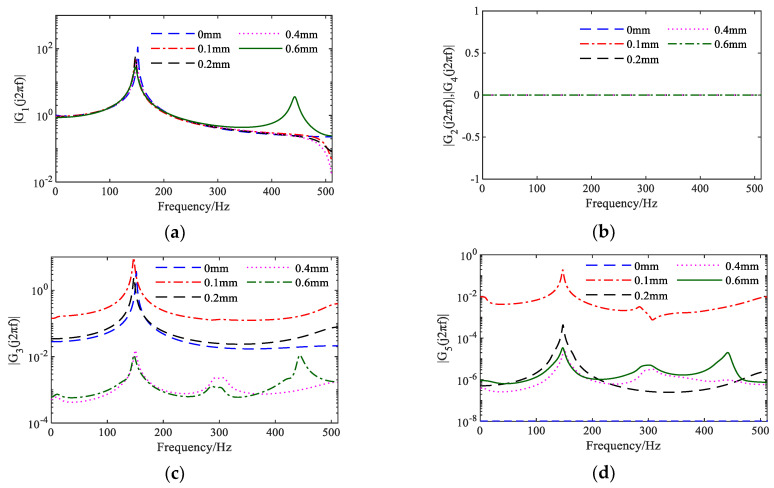
First five orders of NOFRFs: (**a**) first-order NOFRFs; (**b**) second- and fourth-order NOFRFs; (**c**) third-order NOFRFs; (**d**) fifth-order NOFRFs.

- Step 3: Calculate the NOFRFs of the NARX model:

The first five orders of NOFRFs for the identified NARX model are computed based on the GALEs theory, as illustrated in Figure 12. Notably, the second and fourth order NOFRFs are 0, which is attributed to the presence of only odd-order nonlinear terms in the identified NARX model.

**Figure 13:**
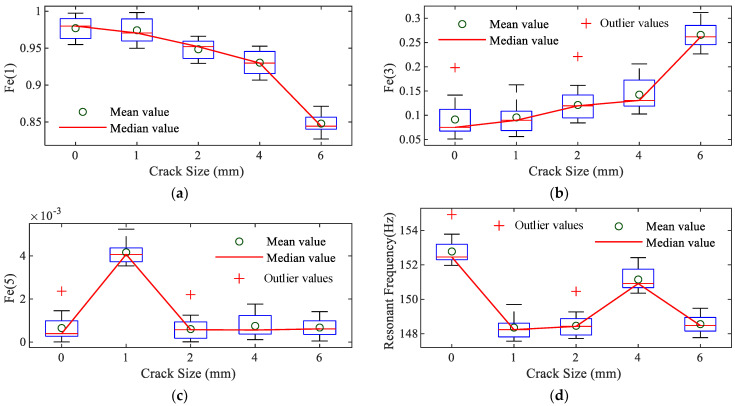
Trends in systemic indicators: (**a**) Fe (1); (**b**) Fe (3); (**c**) Fe (5); (**d**) resonant frequency.

- Step 4: System condition evaluation:

The nonlinear index Fe(n) is calculated for specimens with varying crack depths. To enhance the reliability of the experimental results, steps a to d are repeated, and each group of specimens with different crack depths is tested five times. Figure 13 presents the nonlinear indices Fe(1), Fe(3), and Fe(5), derived from the analysis of 10 sets of experimental data.

As illustrated in Figure 13, the nonlinear indices Fe(1) and Fe(3) exhibit a monotonic relationship with increasing crack depth, whereas Fe(5) does not demonstrate a consistent trend. For comparison with traditional inspection methods, which rely on changes in the first-order natural frequency, the first-order natural frequency of beams with varying crack depths was also measured during the experiments. The results reveal that the change in natural frequency does not vary monotonically with increasing crack depth, which verifies the advantages of the structural damage detection based on NOFRFs.

## 5. Conclusions

In this study, to enhance the accuracy of NARX models and reduce the influence of noise interference effects, we propose an MFROLS algorithm for identifying NARX models of structural systems. Subsequently, numerical simulations were conducted on a one-dimensional multi-degree-of-freedom system using a combined NARX-GALEs approach. By introducing nonlinear stiffness terms to simulate structural damage and comparing the results with conventional LSM, our method successfully captured dynamic characteristic variations under different nonlinear stiffness conditions, while the LSM cannot do so. Experimental validation was performed on simply supported beams with varying damage severities. The results demonstrate that the proposed nonlinear frequency-domain analysis can effectively identify damage-induced variations in beam structures. However, the proposed methodology has two main limitations: (1) it needs to rely on the input excitation of the system, which may not be realized in some engineering practices; and (2) in the process of NARX model identification, the identification process needs to be repeated several times until a result that meets the model prediction output MPO accurately is found, which greatly limits the application of this method, so in the future, these current problems can be considered to improve from the perspective of multi-input and multi-output system identification as well as frequency-domain analysis.

## Figures and Tables

**Table 1:** VAF values of the MPO (time domain) of the discrimination results for different excitations.

Signal Type	Single Harmonic Excitation	Shock Excitation
ARX-a	99.81%	98.92%
ARX-b	99.95%	99.23%
NARX-a	99.64%	99.30%
NARX-b	99.57%	99.37%

**Table 2:** VAF values of MPO for recognized results at different crack depths.

VAF	0 mm	1 mm	2 mm	4 mm	6 mm
NARX-1	99.75%	99.02%	99.69%	99.52%	99.19%
NARX-2	99.78%	98.57%	99.64%	94.52%	99.17%

## Data Availability

No new data were created or analyzed in this study. Data sharing is not applicable to this article.

## Appendix A

The core principle of the GALEs method lies in its ability to analyze and interpret the behavior of nonlinear systems by decomposing them into a series of linear subsystems. Each subsystem corresponds to a distinct order of response within the nonlinear system, facilitating a more accurate and systematic evaluation of the system’s behavior across different frequency ranges. This approach provides a deeper understanding of the nonlinear dynamics and enhances the ability to characterize complex system responses.

GALEs of the NARX model Equation (4) can be derived from using the recursive algorithm proposed in Refs. [15,23].

(A1) $$\begin{array}{l} y_{n}(k)-\sum_{k_{1}=1}^{K}C_{1,0}(k_{1})y_{n}(k-k_{1})=\sum_{k_{1},k_{n}=1}^{K}C_{0,n}(k_{1},\cdots ,k_{n})\prod_{i=1}^{n}u(k-k_{i})\\ \;+\sum_{q=1}^{n-1}\sum_{p=1}^{n-q}\sum_{k_{1},\;k_{p+q}=1}^{K}C_{p,q}(k_{1},\cdots ,k_{p+q})y_{n-q,p}^{K_{p+q}}(k)\prod_{i=p+1}^{p+q}u(k-k_{i})\\ \;+\sum_{p=2}^{n}\sum_{k_{1},\;k_{p}=1}^{K}C_{p,0}(k_{1},\cdots ,k_{p})y_{n,p}^{K_{p}}(k) \end{array}$$

where *n* ≥ 1, $K_{n}$ $=(k_{1},\ldots ,k_{n})$,

(A2) $$\left\{\begin{array}{l} y_{n,p}^{K_{p}}(k)=\sum_{i=1}^{n-(p-1)}y_{i}(k-k_{p})y_{n-i,p-1}^{K_{p}}(k)\\ y_{n,1}^{K_{1}}(k)=y_{n}(k-k_{1}) \end{array}\right.$$

Simple steps to evaluate NOFRFs using GALEs: First, the nth-order output response of the NARX model, as defined in Equation (4), is calculated according to Equation (A1).

(A3) $$\begin{array}{l} y_{n}(k)=\sum_{k_{1}=1}^{K}C_{1,0}(k_{1})y_{n}(k-k_{1})+\sum_{k_{1},k_{n}=1}^{K}C_{0,n}(k_{1},\cdots ,k_{n})\prod_{i=1}^{n}u(k-k_{i})\\ +\sum_{q=1}^{n-1}\sum_{p=1}^{n-q}\sum_{k_{1},\;k_{p+q}=1}^{K}C_{p,q}(k_{1},\cdots ,k_{p+q})y_{n-q,p}^{K_{p+q}}(k)\prod_{i=p+1}^{p+q}u(k-k_{i})+\sum_{p=2}^{n}\sum_{k_{1},\;k_{p}=1}^{K}C_{p,0}(k_{1},\cdots ,k_{p})y_{n,p}^{K_{p}}(k) \end{array}$$

Then, the nth order input spectrum and output spectrum are calculated by Fourier Transform as in Equation (7).

(A4) $$Y_{n}(j\omega )=G_{n}(j\omega )U_{n}(j\omega )$$

The NOFRFs of the nonlinear system at each order are ultimately determined using Equation (10). As a result, the NOFRFs of the NARX model can be derived from the GALEs by calculating the nth-order output spectra $Y_{n}$(j*ω*) and the input spectra $U_{n}$(j*ω*) for *n* = 1, …, *N*. Moreover, Zhu et al. [23] have developed an efficient computational algorithm for GALEs tailored to nonlinear systems, facilitating faster and more accurate calculations.
